# Global Analysis of Tracking the Evolution of SARS-CoV-2 Variants

**DOI:** 10.3390/vaccines11121812

**Published:** 2023-12-04

**Authors:** Muhammad Atif Zahoor

**Affiliations:** Toronto Center for Liver Disease, University Health Network, University of Toronto, Toronto, ON M5G 1L7, Canada; atif.zahoor@uhn.ca

Severe acute respiratory syndrome coronavirus 2 (SARS-CoV-2), infamously known as Coronavirus Disease 2019 (COVID-19), is responsible for the current pandemic and, to date, has greatly impacted public health and economy globally. As of 30 November 2023, out of 722 million confirmed cases, approximately seven million people have died worldwide (https://covid19.who.int). SARS-CoV-2 is a β-coronavirus (CoVs) and belongs to the *Coronaviridae* family. Members of this family are large, enveloped, positive-strand RNA viruses which can be further divided into four genera, i.e., Alpha, Beta, Gamma, and Delta. Alpha and Beta CoVs are known to infect humans and include HCoV229E, NL63, OC43, and HKU01. These are endemic globally and can account for 10–30% of upper respiratory tract infections in adults. High-pathogenic CoVs, such as SARS-CoV, Middle East respiratory syndrome coronavirus (MERS-CoV), and SARS-CoV-2, can cause severe and often life-threatening disease in humans [1]. CoVs are ecologically diverse, with the greatest variety seen in bats, suggesting that they are the reservoirs of many of these viruses [2].

The SARS-CoV-2 genome consists of approximately 30 kb in length and encodes four structural (nucleocapsid (N), membrane (M), envelope (E), and spike (S)), sixteen non-structural, and nine accessory proteins. The glycosylated S protein is located on the surface of the virion and contains the receptor binding domain (RBD) which mediates binding to the angiotensin converting enzyme 2 (ACE2) receptor. The structural proteins play a role in virion formation, assembly, and translation, whereas the non-structural proteins play an essential role in viral replication and transcription. In contrast, nine accessory proteins perform a diverse range of functions, including viral release and inhibition of the host immune response [3]. SARS-CoV-2 possesses an error-prone viral RNA-dependent RNA polymerase with limited 3′-5′ exoribonuclease activity, giving rise to progeny viruses with new mutations which, upon new infection, can exhibit increased transmissibility, increased virulence, and reduced susceptibility to vaccines or therapeutics [3]. 

Over the course of the pandemic, several variants have emerged with mutations in the spike protein and have shown increased virulence and transmission, e.g., the three most notable variants of concern (VOCs) originating from South Africa (B.1.351 as Beta), Brazil (P.1 as Gamma), and India (B.1.617.2 as Delta) [4]. In addition, several variants of interest (VOIs) have been reported, including B.1.525 as eta, B.1.526 as iota, B.1.427/B.1.429 as Epsilon, B.1.617.1 as Kappa, C.37 as Lambda, and B.1.621 as Mu [5]. In November 2021, a variant of the SARS-CoV-2 coronavirus emerged, named as Omicron, which now accounts for over 98% of the publicly available sequences since February 2022. The emergence of new mutations, either derived from these previously circulating VOCs/VOIs or completely new variants, remains possible [6].

Thanks to collective efforts being made by the scientific community, as of 19 November 2023, 13 billion vaccines doses have been administered. Although vaccine candidates are promising, there is still a concern towards assessing their efficiency with respect to emerging variants [7]. In the past, the time taken from a positive PCR diagnosis to sequence results have favored the variants not only in establishing the disease but also in their further spread. However, the most recent measure of completely stopping PCR testing and reliance on antigen testing kits could possibly lead to potential under-reporting of emerging variants. Such a scenario will likely lead to a variant crisis in the future and could impact the current therapeutic approaches.

The monitoring and tracking of new variants on a global scale, as well as the characterization of mutations, is crucial because of their effect on adaptive immunity, their neutralizing antibody response, and their impact on vaccine efficacy [8,9]. Since public health policy needs up-to-date knowledge of the presence of variants circulating in the local population, as well as a critical review of the key drivers that generate virus diversity [7], we initiated this Special Issue, which mainly focuses on the global tracking of COVID19 variants. The topic is extremely important; thus, only high-quality manuscripts were published.

## An Overview of the Published Articles

Ye et al. (contribution 1) assessed the reinfection risk among people with confirmed COVID19 and explored the effect of hybrid immunity. They found lower reinfection with the Omicron variant among individuals with a previous SARS-CoV-2 infection and those who had a booster vaccination. They concluded that hybrid immunity may offer protection against reinfection with Omicron sublineages.

Alcantra et al. (contribution 2) predicted the antibody efficacy against future Omicron subvariants and conducted deep mutational scanning (DMS) encompassing all single mutations of the receptor-binding domain of the BA.2 strain. They highlighted the potential of DMS as a predictive tool for antibody escape and suggested that detailed characterization could lead to the effective management of future SARS-CoV-2 variants.

Jaiswal et al. (contribution 3) explored the role of mycobacterium-*w* (Mw) in boosting adaptive natural killer (ANK) cells and protection against COVID-19 in healthcare workers and concluded that prior exposure to Mw can provide robust protection against the Delta variant of SARS-CoV-2, even with limited ChAdOx1 nCoV-19 vaccination.

Hammad et al. (contribution 4) investigated the maintenance of antibody response of the Oxford–AstraZeneca vaccine (ChAdOx1/nCoV-19) in healthcare workers (HCW). They reported that the vaccine is generally safe and provided a highly effective long-term humoral immune response against the Delta and Omicron variants of SARS-CoV-2. They further suggested that the measurement of serum anti-spike IgG levels before and after vaccination doses can be used as a standardized tool for predicting vaccine immunoreactivity.

Scotto et al. (contribution 5) conducted a prospective study to evaluate the effectiveness of either casirivimab/imdevimab, sotrovimab, or bamlanivimab/etesevimab mAbs against SARS-CoV-2 among patients at risk of severe disease progression. Although their data were significantly important, further studies were suggested to evaluate the real efficacy of mAbs in the treatment of SARS-CoV-2.

## Data Availability

No new data were created or analyzed in this study. Data sharing is not applicable to this article.
